# Impact of the Act‐Belong‐Commit campaign on mental health help‐seeking behaviour

**DOI:** 10.1002/hpja.605

**Published:** 2022-04-26

**Authors:** Catherine F. Drane, Geoffrey Jalleh, Chad Lin, Robert J. Donovan

**Affiliations:** ^1^ National Centre for Student Equity in Higher Education Curtin University Perth WA Australia; ^2^ School of Public Health Curtin University Perth WA Australia; ^3^ School of Human Sciences University of Western Australia Perth WA Australia

**Keywords:** Act‐Belong‐Commit, help‐seeking behaviour, mental health and wellbeing, mental health promotion

## Abstract

**Issue addressed:**

Despite the high prevalence of mental ill‐health amongst Australians, many people do not seek help for their mental ill‐health. A delay in help‐seeking is associated with poorer outcomes. This study investigated the extent to which the Act‐Belong‐Commit mental health promotion campaign prompted people to seek information or professional help for mental ill‐health.

**Methods:**

A sample of 1200 respondents took part in two state‐wide surveys (n = 600 each). Participants aware of the Act‐Belong‐Commit campaign were asked questions related to information‐seeking and help‐seeking behaviours because of the campaign.

**Results:**

Of those aware of the campaign, 8% stated that the campaign prompted them to seek information and 4% stated that the campaign prompted them to seek help for a mental health problem. Those with a mental illness experience (MIE) were significantly more likely than those without to report that the campaign prompted them to look for information (12% vs 6%) and seek help for a mental health problem (9.5% vs 1.2%). Extrapolating these results to the total adult population of Western Australia indicated that around 120 000 adults had sought mental health information, and around 60 000 had sought help as a result of the campaign.

**Conclusions:**

The campaign not only initiated the seeking of information or professional help for a mental health problem amongst those with no prior thoughts of such, but also prompted those who were already thinking about seeking information or getting help to act sooner than they otherwise would.

**So what?:**

Although previous research suggests that mental health literacy interventions have limited impact on help‐seeking, the presented data show that the Act‐Belong‐Commit approach can have a significant impact on help‐seeking, particularly amongst those with a MIE, which could yield substantial social and economic return on investment benefits if intensified at both the media and community grass roots levels.

## 1 INTRODUCTION

Almost half the Australian population (45%) experience some form of mental ill‐health in their lifetime, and one in five Australians experience a mental disorder in any 1 year.^1^ However, despite the high prevalence of mental ill‐health, many people do not seek help to manage their mental ill‐health. For many people with poor mental health, communicating their difficulties to others to seek help and support, can be challenging.^2^ However, a delay in seeking help and treatment is associated with poorer health outcomes, such as long‐term mental ill‐health, increased risk of harmful behaviours and the development of other mental disorders.^3^ On the other hand, early interventions and support can improve mental health prognoses.^4^

Research has identified several barriers to seeking help, such as perceived stigma, poor mental health literacy, lack of knowledge of available resources and a lack of support from others.^4^, ^5^ Mental health promotion campaigns that address these barriers could be beneficial in changing help‐seeking attitudes and intentions, and hence increase help‐seeking behaviours.

Whilst campaigns specifically targeting people experiencing mental health difficulties can be effective in stimulating them to seek help (eg, European Alliance Against Depression; Beyondblue), and intensive community‐based mental health promotion can increase positive attitudes towards help‐seeking,^6^ there is a lack of information as to whether population‐wide mental health promotion impacts help‐seeking behaviour.^7^

## 2 MENTAL HEALTH PROMOTION

Mental health issues are costly to society,^8^ with an estimated cost to the Australian economy of around $220 Billion per year.^9^ The economic case for prevention and early intervention is compelling and there is increasing attention around the globe to public mental health promotion and illness prevention,^7^, ^10^ and particularly as a result of the COVID pandemic.^11^ There are primary prevention (promotion of protective factors) and mental health literacy programs in settings such as schools and worksites, and community‐based mental health promotion initiatives in geographically limited or remote areas, such as described in Barry (2005).^12^ However, the Act‐Belong‐Commit mental health promotion campaign, and the “ABC of Mental Health” extension in Denmark, the Faroe Islands and Norway, appears to be the only comprehensive, population wide campaign emphasising promotion and primary prevention, while also impacting secondary and tertiary prevention.^13^, ^14^, ^15^

The words “Act,” “Belong” and “Commit,” represent the three behavioural domains that the scientific literature confirms, and that lay people believe, are the behaviours that contribute to positive mental health. These are articulated as follows^16^:

Act: keep physically, mentally, spiritually and socially active: “do something”
Belong: keep up friendships, engage in group activities, participate in community events: “do something with someone”
Commit: set goals and challenges, engage in activities that provide meaning and purpose in life, including taking up causes and volunteering to help others: “do something meaningful—something that matters”

Overall, the Act‐Belong‐Commit message encourages people to be physically, spiritually, socially, and mentally *act*ive in ways that increase their sense of *belong*ing to the communities in which they live, work, play and recover, and that involve *commit*ments to causes or challenges that provide meaning and purpose in their lives.^16^ As noted above there is substantial and increasing evidence that the three Act, Belong and Commit behavioural domains contribute to increasing levels of both physical health and mental health^14^ and act as protective factors against mental disorders, such as depression and anxiety, and cognitive impairment.^17^, ^18^ Furthermore, the three Act‐Belong‐Commit domains appear universal across cultures, although the articulation and emphasis of each of the domains may vary between cultures. For example, not all cultures conceptualise good mental health and health‐promoting factors in precisely the same way, therefore culture specific adaptations may be required when articulating the three domains. Nonetheless, the overall understanding of what constitutes good mental health across cultures remains consistent with the underlying Act‐Belong‐Commit messages.^19^

Given that health literacy relates to “how people access, understand and use health information in ways that benefit their health,”^20^ one of the initial—and continuing—major aims of the Act‐Belong‐Commit and the ABC for Mental Health campaigns is to improve mental health literacy, particularly in terms of informing people of what they can and should do to keep mentally healthy. In that sense, the campaign aims to not only increase openness in talking about mental health and destigmatising mental illness,^13^ but also to stimulate people to undertake activities conducive to good mental health.

In Western Australia, where the Act‐Belong‐Commit campaign originated, the campaign is supported by paid mass and targeted media advertising, including online channels, and through partnerships with a wide variety of community organisations.^21^ The campaign has achieved around 80% awareness in the general population. Annual state‐wide surveys via computer assisted telephone interviews (CATIs) have been conducted to assess people's awareness and understanding of the campaign messages, and whether respondents have “tried to do something for their mental health” as a result of their exposure to the campaign messages. These CATI participants have shown a good understanding of the campaign messages, with almost all nominating behaviours under one or more of the Act, Belong and Commit domains when asked “what they thought the campaign was trying to do,” and, amongst those aware of the campaign, around 10%‐15% have reported doing something to improve their mental health in response to the campaign.^13^ When asked what they did, as expected, the majority of responses to this open‐ended question fell into the three Act‐Belong‐Commit behavioural domains.

Although the above question relates to whether respondents had tried to do the sorts of behaviours promoted by the campaign messages (which do not include “help‐seeking for a mental health problem”), a small number of respondents have spontaneously reported that they sought professional help. A low reporting of help‐seeking in response to that question is not unexpected given the question wording. Also, as noted above, unlike mental illness campaigns that urge their target audiences to seek professional help, the Act‐Belong‐Commit campaign media messages focus only on encouraging the target audience to engage in activities that are conducive to good mental health. Given these spontaneous responses, specific questions were included in the CATI questionnaire in 2018 and 2019 to quantify the extent to which the campaign prompted people to seek information about, or professional help for, mental health problems. Those who indicated that they had sought information or professional help were asked whether the campaign *initiated* these actions or whether they were already considering these actions and the campaign prompted them to act *sooner* than they otherwise would have. Previous evaluation research has shown that a significantly higher proportion of respondents with a mental illness experience (MIE) reported trying to “do something to be more mentally healthy” as a result of the campaign, compared to those not reporting a MIE.^15^ Therefore, the current study analysed the help‐seeking data by whether or not respondents reported a MIE.

## 3 METHOD

### 3.1 Sample

A total of 1200 adult respondents took part in the two CATI surveys (n = 600 in each year). Quotas were set such that 50% were female and 50% were male and all age groups represented (55% were 18‐49 years and 45% were 50 years and over.^13^, ^15^ Of these 1200 respondents, almost 80% (n = 933) reported being aware of the campaign and were asked the following information‐seeking and help‐seeking questions.

### 3.2 Information‐seeking questions

Respondents aware of the campaign were asked whether “thinking about the Act‐Belong‐Commit campaign has prompted you to look for or make enquiries about where you could get information about mental health and mental health problems?” Those who answered “yes” to this question were provided with the following response categories and asked which—if either—applied to them: “I was already thinking about looking for information and the campaign prompted me to do it sooner than I would have” or “I wasn't thinking about looking for information, but the campaign prompted me to.”

### 3.3 Help‐seeking questions

Respondents aware of the campaign were then asked whether “thinking about the campaign prompted you to actually get help for a mental health problem?” Those who answered “yes” to this question were provided with the following response categories and asked which—if either—applied to them: “I was already thinking about getting help and the campaign prompted me to do it sooner than I would have” or “I wasn't thinking about getting help, but the campaign prompted me to.” Those stating they had sought help were also asked “from where they got help.”

Half were asked these questions in the context of “over the past year or so” and half were asked in the context of “ever” (Given the relatively small numbers involved, the results for “the past 12 months” and “ever” are combined for this report). All participants were also asked whether they had “ever been diagnosed with a specific mental illness” and whether “in the past 12 months or so they had seen a counsellor, doctor or psychologist because of a mental health problem.” Those reporting either, were classified as having a “mental illness experience.” Approximately equal proportions of males and females reported a MIE.

## 4 RESULTS

Of the n = 933 respondents aware of the Act‐Belong‐Commit campaign, Table 1 shows that 8% (n = 74) stated that the campaign had prompted them to *seek information* about mental health and mental health problems, and 4% (n = 35) stated that the campaign had prompted them to *seek help* for a mental health problem. Further analysis showed that 9.4% (n = 88) reported that thinking about the campaign had prompted them to *either* seek information *or* to seek help for a mental health problem. There were no differences in seeking help or seeking information by gender. However, younger respondents were more likely to seek information (12% of 18‐39‐year‐olds vs 5% of 40 years and older), and to seek help (5% of 18‐39‐year‐olds vs 2% of 40 years and older).

**TABLE 1:** Percent of those aware of the campaign who sought information or help by mental illness experience (MIE)

Action	% total aware (n = 933)	% reporting MIE (n = 284)	% not reporting MIE (n = 649)
Sought information	8.0	12.0	6.2
Sought help	3.8	9.4	1.2

Of the n = 35 who sought help, just over half (n = 20) nominated a mental health professional and just over a third (n = 13) nominated a GP (one nominated a “friend” and one did not specify a source).

Table 1 shows that significantly and substantially greater proportions of those with a MIE vs the rest of the sample, reported that the campaign prompted them to look for information: 12% vs 6% (*P* = .004); and, perhaps more importantly, to seek help for a mental health problem: 9.4% vs 1.2% (*P* = .000).

Whilst these percentages might appear small, extrapolating these results to the total adult population in Western Australia indicates that at the time of the surveys, around 120 000 adults had sought information about mental health as a result of the campaign, and around 60 000 had sought help for a mental health problem (based on an estimated total population of 2.7 m, with approximately 80% aged 18+,^22^ and almost 80% aware of the campaign [as above]). These numbers provide a concrete indication of the potential benefits of the A‐B‐C campaign in stimulating help‐seeking behaviours in the adult general population.

With respect to whether the Act‐Belong‐Commit campaign *initiates* or simply *brings forward* an existing intention to seek information or help about a mental health problem, Table 2 shows that the campaign stimulates both types of action, with information‐seeking slightly more likely to be *initiated* than *brought forward* (46% vs 32%, respectively), whereas with help‐seeking, equal proportions stated that the campaign *initiated* that behaviour vs prompting them to *act sooner* (37% for both).

**TABLE 2:** Whether the campaign initiated seeking behaviour or prompted acting sooner

Initiated action or prompted sooner	% seeking information (N = 74)	% seeking help (N = 35)
Campaign *initiated* my action	46	37
Campaign prompted me to act *sooner* than otherwise	32	37
Neither/Don't know	22	26
Total	100	100

## 5 DISCUSSION

A recent review of public mental health interventions found that mental health literacy interventions have limited impact on help‐seeking.^7^ However, extrapolating the above help‐seeking percentages to the total Western Australian adult population suggests that the Act‐Belong‐Commit approach can have a significant impact on help‐seeking, whether for information about mental health problems or for professional help. Given that around two‐thirds of respondents aware of the Act‐Belong‐Commit campaign believe that the campaign has increased openness about mental health issues and decreased stigma around mental illness,^23^ it is likely that the campaign's impact in these areas has reduced these barriers to help‐seeking. This stigma reduction, in conjunction with encouraging people with a mental illness to take up mental health promoting activities is also consistent with the increasing focus on “indirect” ways of treating and preventing mental illnesses, and particularly depression.^24^

The data suggest that the campaign not only initiates the seeking of information or getting professional help for a mental health problem amongst those with no prior thoughts of such, but also prompts those who are already thinking about seeking information or getting help to act sooner than they otherwise would. Furthermore, a substantially greater proportion of those with a MIE report seeking information about or professional help for a mental health problem as a result of the campaign. These are important findings given the importance of early intervention.^4^ Given that the majority of those who seek professional help do so from a mental health professional or GP, Act‐Belong‐Commit should consider developing targeted materials to increase knowledge and understanding of the campaign in these professions, perhaps as part of a “social prescribing” or “lifestyle medicine” approach,^14^, ^25^ and particularly as help seekers are likely to mention the campaign as stimulating their help‐seeking behaviour.

As noted above, previous data on whether respondents have tried to do something to be more mentally healthy as a result of the Act‐Belong‐Commit campaign have shown that in addition to having a primary prevention role, the campaign also plays secondary and tertiary prevention roles, in that a significantly higher proportion of respondents with a MIE report trying to “do something to be more mentally healthy” as a result of the campaign vs those not reporting a MIE.^15^ This study supports those findings in that those with a MIE were far more likely than those not reporting such to seek information as a result of the campaign, and perhaps more importantly, to actually seek help for a mental health problem, thus confirming a tertiary prevention effect.

Overall, these data confirm that Act‐Belong‐Commit contributes to primary, secondary and tertiary prevention, and supports the proposition that the campaign would yield substantial social and economic return on investment benefits^26^ if intensified at both the media and community grass roots levels, and if there were closer cooperation and collaboration with general practitioners, psychologists, psychiatrists and mental health services in general.

## CONFLICT OF INTEREST

The authors declare no conflict of interest.

## ETHICS STATEMENT

The project received ethics approval from Curtin University's Human Research Ethics Committee (RDHS‐235‐15).

## References

[1] Australian Bureau of Statistics . National Health Survey: first results, 2017‐18. ABS Cat. No. 4364.0.55.001. Canberra: Australian Bureau of Statistics. Available from: https://www.abs.gov.au/ausstats/abs@.nsf/mf/4364.0.55.001

[2] Rickwood D , Thomas K , Bradford S . Review of help‐seeking measures in mental health: a evide4nce check rapid review. Sax Institute; 2012; p. 1–35. Available from: https://www.saxinstitute.org.au

[3] Cheung R , O'Donnell S , Nawaf M , Elliot MG . Factors associated with delayed diagnosis of mood and/or anxiety disorders. Health Promot Chronic Dis Prev Can. 2017;37(5):137. Available from: 10.24095/hpcdp.37.5.02–48. 28493658 PMC5650019

[4] Gulliver A , Griffiths KM , Christensen H , Brewer JL . A systematic review of help‐seeking interventions for depression, anxiety and general psychological distress. BMC Psychiatry. 2012;12(1):1–2. Available from: http://www.biomedcentral.com/1471-244X/12/81 22799879 10.1186/1471-244X-12-81 PMC3464688

[5] Salaheddin K , Mason B . Identifying barriers to mental health help‐seeking among young adults in the UK: a cross‐sectional survey. Br J Gen Pract. 2016;66(651):686–92. 10.3399/bjgp16X687313 PMC5033305 27688518

[6] Reynolds C , Byrne M , Barry M . Final evaluation report of the rural mental health project (phase 2). NUI, Galway: Centre for Health Promotion Studies; 2004.

[7] Campion J . Public mental health: evidence practice and commissioning. London: Royal Society for Public Health; 2019.

[8] Christensen MK , Lim CC , Saha S , Plana‐Ripoll O , Cannon D , Presley F , et al. The cost of mental disorders: a systematic review. Epidemiol Psychiatr Sci. 2020;29:e161. 10.1017/S204579602000075X 32807256 PMC7443800

[9] Productivity Commission . Mental health. Report no. 95, Canberra; 2020.

[10] Arango C , Díaz‐Caneja CM , McGorry PD , Rapoport J , Sommer IE , Vorstman JA , et al. Preventive strategies for mental health. Lancet Psychiatry. 2018;5(7):591–604. 29773478 10.1016/S2215-0366(18)30057-9

[11] International Union for Health Promotion and Education . Position statement on critical actions for mental Health promotion. Paris: IUPE; 2021 Available from: https://www.iuhpe.org/images/IUHPE/Advocacy/IUHPE_Mental-Health_PositionStatement.pdf

[12] Barry M . Creating a mentally healthy society: lessons from a cross‐border rural mental health project in Ireland. J Public Ment Health. 2005;1:30–4. Available from. 10.1108/17465729200500008

[13] Anwar‐McHenry J , Donovan RJ , Jalleh G , Laws A . Impact evaluation of the Act‐Belong‐Commit mental health promotion campaign. J Public Ment Health. 2012;11(4):186–95.

[14] Donovan RJ , Anwar‐McHenry J . Act‐Belong‐Commit: a lifestyle‐related mental health program that might also work for clinicians. Am J Lifestyle Med. 2014;10(3):193–9. 10.1177/1559827614536846 30202274 PMC6124955

[15] Donovan R , Jalleh G , Robinson K , Lin C . Impact of a population‐wide mental health promotion campaign on people with a diagnosed mental illness or recent mental health problem. Aust N Z J Public Health. 2016;40(3):274–5. 27028403 10.1111/1753-6405.12514

[16] Koushede V , Donovan RJ . The application of Salutogenesis to communitywide mental health promotion: the Act‐Belong‐Commit/ABC's of Mental Health Campaign and Framework. In: Mittelmark M , editor. The handbook of Salutogenesis. 2nd ed. Cham: Springer; 2021.

[17] Santini ZI , Koyanagi A , Tyrovolas S , Haro JM , Donovan RJ , Nielsen L , et al. The protective properties of Act‐Belong‐Commit indicators against incident depression, anxiety, and cognitive impairment among older Irish adults: findings from a prospective community‐based study. Exp Gerontol. 2017;91:79–87. Available from:. 10.1016/j.exger.2017.02.074 28257931

[18] Santini ZI , Nielsen L , Hinrichsen C , Tolstrup JS , Vinther JL , Koyanagi A , et al. The association between Act‐Belong‐Commit indicators and problem drinking among older Irish adults: findings from a prospective analysis of the Irish longitudinal study on ageing (TILDA). Drug Alcohol Depend. 2017b;180:323–31. 28950238 10.1016/j.drugalcdep.2017.08.033

[19] Nielsen L , Sørensen BB , Donovan RJ , Tjørnhøj‐Thomsen T , Koushede V . ‘Mental health is what makes life worth living’: an exploration of lay people's understandings of mental health in Denmark. Int J Ment Health Promot. 2017;19(1):26–37. 10.1080/14623730.2017.1290540

[20] Australian Institute of Health and Welfare (AIHW). Health literacy; 2020. Available from: https://www.aihw.gov.au/reports/australias-health/health-literacy.

[21] Donovan RJ . Social franchising approaches for Community‐Based Mental Health Promotion. In: Fourali C , French J , editors. The Palgrave encyclopedia of social marketing. Cham: Palgrave Macmillan; 2021. 10.1007/978-3-030-14449-4_67-1

[22] Australian Bureau of Statistics . National, state and territory population. Australian Bureau of Statistics: Canberra; 2021 Available from: https://www.abs.gov.au/statistics/people/population/national-state-and-territory-population/mar-2021

[23] Lin C , Jalleh G , Donovan RJ . Evaluation of the Act‐Belong‐Commit Mentally Healthy WA Campaign: 2019 survey data. Perth: Behavioural Research Group, School of Public Health, Faculty of Health Sciences, Curtin University; 2018.

[24] Cuijpers P . Indirect prevention and treatment of depression: an emerging paradigm? Clin Psychol Eur. 2021;3(4):1–9. 10.32872/cpe.6847 PMC9667226 36398290

[25] Nielsen L , Hinrichsen C , Nelausen MK , Santini Z , Meilstrup CB . ABC for mental health—a tool for better wellbeing. Månedsskrift for Almen Praksis: Denmark; 2021.

[26] McDaid D , Park AL , Wahlbeck K . The economic case for the prevention of mental illness. Annu Rev Public Health. 2019;40:373–89. 10.1146/annurev-publhealth-040617-013629 30601725

